# Effects of changes in occupational stress on the depressive symptoms of Korean workers in a large company: a longitudinal survey

**DOI:** 10.1186/s40557-018-0249-5

**Published:** 2018-06-08

**Authors:** Jaehyuk Jung, Inchul Jeong, Kyung-Jong Lee, Guyeon Won, Jae Bum Park

**Affiliations:** 1 Department of Occupational and Environmental Medicine, Ajou University Hospital, Suwon, South Korea; 2 Department of Occupational and Environmental Medicine, Ajou University School of Medicine, 164 World cup-ro, Yeongtong-gu, Suwon, 16499 South Korea

**Keywords:** Occupational stress, Depression, Korean workers, Korean occupational stress scale

## Abstract

**Background:**

Prospective studies on occupational stress and depression among Korean workers are scarce. This study aimed to investigate the causal relationship between changes in occupational stress and the presence of depression.

**Methods:**

This study analyzed data from a survey conducted with workers in a large Korean company (2015–2016). Occupational stress was measured using the Korean Occupational Stress Scale, and depression was screened using the WHO-5 Well-being Index. The levels of occupational stress were grouped in quartiles based on subjects’ occupational stress scores in 2015, and changes in occupational stress were measured using the score changes between the 2015 and 2016 surveys. Subjects were divided into four groups according to the presence or absence of depression in 2015 and 2016: a non-depressed group whose mental health did not change, a non-depressed group whose mental health deteriorated, a depressed group that continued to be depressed, and a depressed group whose symptoms of depression were alleviated. Logistic regression analysis was used to calculate the odds ratios (ORs) and confidence intervals (CIs) of the subjects’ deterioration in mood or alleviation of depression by occupational stress levels and changes.

**Results:**

The OR for developing depressive symptoms was 2.51 (95% CI 1.46–4.33) and 2.73 (95% CI 1.39–5.36) in the third and the fourth quartiles of occupational stress, respectively, compared to the first quartile. When the occupational stress score increased by 1 point, the OR for increasing depressive symptoms was 1.07 (95% CI 1.04–1.10). There was no significant difference in the alleviation of depressive symptoms between the groups by level of occupational stress. However, when the occupational stress score decreased by 1 point, the OR for alleviating depressive symptoms was 1.08 (95% CI 1.05–1.11).

**Conclusion:**

The results showed that increased occupational stress increased the presence of depressive symptoms, and that reduced occupational stress suppressed the development of depressive symptoms. More attention should be paid to stress management among workers.

## Background

Occupational stress occurs during the performance of one’s duties and is defined as harmful physical and emotional responses that occur when job requirements do not match a worker’s capabilities, resources, or demands [1]. Therefore, although there may be some differences in occupational stress levels, depending on types of work required of individuals, all humans who work experience physiological effects arising from stress.

As workers’ health problems, such as industrial incidents and occupational diseases, have recently become important, occupational stress, which may also damage workers’ health, has also received attention as a risk factor. Mental-health problems, such as depression [2] and sleep disorders [3], as well as physical illnesses, such as cardiovascular [4] and musculoskeletal diseases [5], are also known to be associated with occupational stress. In particular, mood disorders, such as depression, are common in adult populations, including workers. In Europe, the one-year prevalence of depression is 6.9% [6]. In South Korea, the estimated lifetime prevalence and one-year prevalence of major depression is 5.0 and 1.5%, respectively, which indicates that the number of people who have experienced depression during the previous year is estimated to reach 610,000. It has also been reported that 15.4% of adults have seriously considered suicide more than once in their lifetime [7].

Therefore, there has been a growing interest in occupational stress and depression, and many studies on this topic have been conducted since the Korean Occupational Stress Scale was developed. Increases in relationship conflicts and problems related to organizational culture have been reported to be associated with a higher risk of depression, and 1 out of 10 workers has a high likelihood of suffering from depression [8]. Additionally, a variety of studies have found that among small and medium enterprise workers, male white-collar workers in automobile companies, immigrant workers, female workers in traditional markets, and dentists have higher occupational stress, which is associated with a higher risk of depressive symptoms [9–13]. However, none of those studies have revealed causal relationships between their independent and dependent variables due to the limitations of the studies’ cross-sectional designs; thus, the need for longitudinal studies is raised frequently. Although studies have used explanatory models to examine occupational stress [14, 15], these studies are difficult to be applied straightforwardly in Korea on account of not targeting Korean workers and without considering Korean occupational climate. Therefore, longitudinal study using Korean occupational stress scale is needed.

This prospective study was intended to investigate whether changes in (i.e., greater or less) occupational stress significantly affects the risk for developing depressive symptom in addition to that occupational stress influences the risk for depression. The results of this study can be used: (a) to emphasize the importance of occupational stress management in the prevention of mental-illnesses among workers, (b) as evidence for reducing the prevalence of depression through workers’ effective stress management, and (c) as knowledge to help improve workers’ health and corporate productivity more efficiently when selecting and managing employees who are vulnerable to depression.

## Methods

### Subjects

The study subjects were workers in the company who participated in a health examination and a survey from May to July in both 2015 and 2016. The company is a single large company manufacturing home electronic appliance such as TV, monitor, and smartphone. The company was located in Gyeonggi-do, South Korea. A total of 1037 workers underwent health examinations in the two consecutive years, and data from 1036 subjects were analyzed for this study; data submitted by one worker with missing values were excluded. The survey included information pertaining to workers’ personal characteristics, work-related characteristics, and scores on the Korean Occupational Stress Scale-Short Form (KOSS-SF) and the WHO-5 Well-being Index.

### Variables and instruments

#### Sociodemographic and work-related characteristics

The general characteristics of the subjects included sex, age, smoking, drinking, exercise, marital status, and educational background. For drinking status, excessive drinking was defined as having more than 14 drinks per week for male adults and having more than 7 drinks per week for female adults. Performing physical activity with moderate or higher intensity for more than 150 min per week was defined as appropriate physical activity according to the Physical Activity Guidelines for Koreans issued by the Ministry of Health and Welfare Affairs. Work-related characteristics that were measured included the number of years of service, number of hours worked per week, and occupational classification, which included white-collar workers in research and development department, blue-collar workers in production department, and service workers in sales department.

#### Occupational stress

Occupational stress was measured using the Korean Occupational Stress Scale-Short Form (KOSS-SF). The KOSS-SF consists of 7 sub-domains with 24 items. The 7 sub-domains include job demand, insufficient job control, job insecurity, interpersonal conflict, organizational system, lack of reward, and occupational climate. Each item is rated on a Likert scale (“strongly disagree,” “disagree,” “agree,” and “strongly agree.”). The total score is calculated by dividing the sum of the converted scores for the 7 sub-domains by 7. A higher score indicates greater occupational stress [16]. In this study, occupational stress level was divided into quartiles based on the data from the four groups in 2015, when this study began. A change in occupational stress was indicated by the difference in the total and 7 sub-domains occupational stress score between year one and year two. The depressed-deteriorated group was calculated by subtracting subjects’ 2015 occupational stress score from the 2016 score, whereas the change for the alleviated group was calculated by subtracting subjects’ 2016 score from the 2015.

### Depressive symptoms

Symptoms of depression were measured using the WHO-5 Well-being Index Questionnaire. This tool consists of five items indicating respondents’ emotional well-being over the previous 2 weeks (e.g., “I have felt in good spirits.”). Each item is rated on a 6-point scale: “all of the time” (5 points), “most of the time” (4 points), “more than half the time” (3 points), “less than half the time” (2 points), “some of the time” (1 point), and “at no time” (0 points). The total possible score ranges from 0 to 25 points, with a score of less than 13 points indicating a high risk for depression [17].

### Study design

**Fig. 1:**
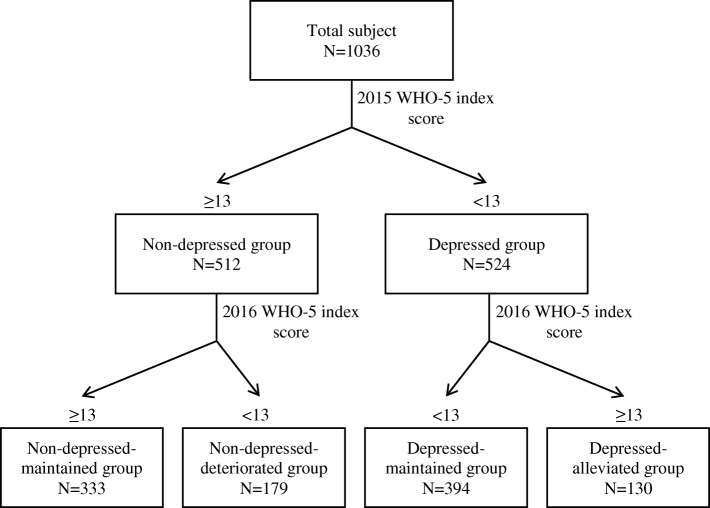
Flow chart of final groups divided by the WHO-5 well-being index score

This prospective study was designed to investigate the effects of occupational stress and changes in occupational stress on changes (deterioration or alleviation) in depression among workers in a large company. Subjects were divided into a non-depressed and a depressed group based on whether they showed an absence or presence of depression in 2015, when the study began. Subsequently, the non-depressed group was divided into a non-depressed group that maintained the absence of depression (non-depressed-maintained group) and a non-depressed group whose mental health deteriorated (non-depressed-deteriorated group)**,** whereas the depressed group was divided into a depressed group that maintained the presence of depression (depressed-maintained group) and a depressed group whose depression was alleviated (depressed-alleviated group) based on the presence or absence of depression in 2016. The purpose of these classifications was to investigate whether occupational stress resulted in the deterioration of subjects’ mental health or alleviation of depression. The flow chart of this study is shown in the Fig. 1.

### Data analysis

The categorical variables were analyzed using the Chi-square test, and the continuous variables were analyzed using the t-test to examine differences in occupational stress levels, general characteristics, and occupational characteristics between the non-depressed-maintained group and the non-depressed-deteriorated group, and between the depressed-maintained group and the depressed-alleviated group.

Logistic regression analysis was used to calculate the odds ratios (ORs) and confidence intervals (CIs) for the effects of occupational stress levels and changes (deterioration or alleviation) in depression of non-depressed-deteriorated group compared with non-depressed-maintained group and depressed-maintained group compared with depressed-alleviated group respectively. The occupational stress change also was independent variable in the same way.

Model I is crude model. The general characteristics of the subjects, including sex, age, smoking, excessive drinking, exercise, educational level, marital status and their occupational classifications including years of service, number of hours worked were included in Model II. Finally, Model III adjusted for occupational stress level in 2015. Total occupational stress score was adjusted while independent variable was total score. Each 2015 sub-domains score was adjusted when independent variable was each sub-domain score likewise.

All statistical analyses were performed using SPSS version 23.0.

## Results

The total study subjects were 1036 workers. The workers tended to be male, non-smokers, non-excessive drinkers, college graduates or lower, unmarried and not engage in adequate physical activity concerning the general characteristics. As for work-related characteristics, the total subjects tended to be white-collar workers and to work 41–50 h per week. The mean age of the subjects was 28.25 years, the mean length of service was 2.26 years.

In 2015, the non-depressed group consisted of 512 subjects, accounting for 49.4% of all subjects and the number of the subjects in the depressed group was 524 (50.6%). There were significant differences in physical activity, continuous service year, working hours, and occupational stress between non-depressed group and depressed group. The depressed group exercised less, had a little bit of a long career, and worked long hours. Typically, the depressed group’s total occupational stress score was 10 points higher than the non-depressed group’s. The significant difference was observed all along the sub-domains as well (Table 1).

**Table 1:** General characteristics of total study subjects between non-depressed group and depressed group at baseline

	Total study subjects (*n* = 1036, 100.0%)	Non-depressed group (*n* = 512, 49.4%)	Depressed group (*n* = 524, 50.6%)	*p*-value
	N (%) or M ± SD	N (%) or M ± SD	N (%) or M ± SD	
Sex				
Male	843 (81.4%)	428 (50.8%)	415 (49.2%)	0.069
Female	193 (18.6%)	84 (43.5%)	109 (56.5%)	
Age (year)				
	28.25 ± 2.82	28.21 ± 2.70	28.30 ± 2.93	0.620
Smoking history				
Non smoker	589 (56.9%)	297 (50.4%)	292 (49.6%)	0.745
Past smoker	158 (15.3%)	75 (47.5%)	83 (52.5%)	
Current smoker	289 (27.9%)	140 (48.4%)	149 (51.6%)	
Heavy drinking				
No	847 (81.8%)	423 (49.9%)	424 (50.1%)	0.478
Yes	189 (18.2%)	89 (47.1%)	100 (52.9%)	
Adequate physical activity				
No	744 (71.8%)	343 (46.1%)	401 (53.9%)	0.001
Yes	292 (28.2%)	169 (57.9%)	123 (42.1%)	
Career (year)				
	2.26 ± 1.32	2.14 ± 1.39	2.37 ± 1.23	0.005
Working hours (hours/week)				
≤ 40	122 (11.8%)	73 (59.8%)	49 (40.2%)	< 0.001
41~ 50	432 (41.7%)	249 (57.6%)	183 (42.4%)	
51~ 60	351 (33.9%)	145 (41.3%)	206 (58.7%)	
≥ 61	131 (12.6%)	45 (34.4%)	86 (65.6%)	
Job task				
Office	886 (85.5%)	445 (50.2%)	441 (49.8%)	0.208
Labor/Service	150 (14.5%)	67 (44.7%)	83 (55.3%)	
Education				
≤ College	840 (81.1%)	417 (49.6%)	423 (50.4%)	0.767
≥ Master degree	196 (18.9%)	95 (48.5%)	101 (51.5%)	
Marital status				
Unmarried/Divorced	793 (76.5%)	384 (48.4%)	409 (51.6%)	0.271
Married	243 (23.5%)	128 (52.7%)	115 (47.3%)	
Occupational stress				
1st quartile	256 (24.7%)	201 (78.5%)	55 (21.5%)	< 0.001
2nd quartile	269 (26.0%)	161 (59.9%)	108 (40.1%)	
3rd quartile	251 (24.2%)	97 (38.6%)	154 (61.4%)	
4th quartile	260 (25.1%)	53 (20.4%)	207 (79.6%)	
Total score	41.72 ± 11.33	36.68 ± 10.30	46.63 ± 10.07	< 0.001
Occupational stress sub-domains score				
Job demand	52.15 ± 17.58	46.50 ± 16.41	57.67 ± 16.95	< 0.001
Insufficient job control	48.32 ± 14.94	45.25 ± 14.11	51.32 ± 15.13	< 0.001
Interpersonal conflict	33.68 ± 14.46	29.56 ± 13.63	37.70 ± 14.12	< 0.001
Job insecurity	35.60 ± 21.94	30.14 ± 20.45	40.94 ± 22.06	< 0.001
Organizational system	43.32 ± 16.70	38.75 ± 14.74	47.77 ± 17.29	< 0.001
Lack of reward	43.85 ± 16.32	37.17 ± 13.73	50.38 ± 16.01	< 0.001
Occupational climate	35.10 ± 17.32	29.41 ± 15.96	40.66 ± 16.79	< 0.001

### Non-depressed group

In 2016, 333 (65.0%) of them remained in the non-depressed-maintained group, whereas 179 (35.0%) experienced deterioration in their mental health and moved to the non-depressed-deteriorated group. In terms of general characteristics, the subjects in this group tended to be male, non-smokers, non-excessive drinkers, college graduates or lower, unmarried, and workers who did not engage in adequate physical activity. As for their work-related characteristics, the subjects in this group tended to be white-collar workers and to work 41–50 h per week. The mean age of the subjects was 28.21 years, the average length of service was 2.14 years, and there were no significant differences in their general or work-related characteristics between non-depressed-maintained group and non-depressed-deteriorated group excluding occupational stress. The proportion of subjects in the lower 25% or first quartile with low occupational stress levels was 74.6% of those who maintained their non-depression status, but the proportion of subjects in upper third and fourth quartiles was as low as 52.6 and 52.8%, respectively. The total score was 35.2 points in the maintained group, and it was significantly lower than those in the group whose mental health deteriorated (39.5 points). The all sub-domains scores of the maintained group were significantly and consistently lower than those of the deteriorated group (Table 2).

**Table 2:** General characteristics of study subjects among non-depressed group at baseline

	Total Non-depressed group (*n* = 512, 100.0%)	Non-depressed-maintained group (*n* = 333, 65.0%)	Non-depressed-deteriorated group (*n* = 179, 35.0%)	p-value
	N (%) or M ± SD	N (%) or M ± SD	N (%) or M ± SD	
Sex				
Male	428 (83.6%)	286 (66.8%)	142 (33.2%)	0.056
Female	84 (16.4%)	47 (56.0%)	37 (44.0%)	
Age (year)				
	28.21 ± 2.70	28.20 ± 2.67	28.22 ± 2.78	0.929
Smoking history				
Non smoker	297 (58.0%)	191 (64.3%)	106 (35.7%)	0.836
Past smoker	75 (14.6%)	51 (68.0%)	24 (32.0%)	
Current smoker	140 (27.3%)	91 (65.0%)	49 (35.0%)	
Heavy drinking				
No	423 (82.6%)	275 (65.0%)	148 (35.0%)	0.978
Yes	89 (17.4%)	58 (65.2%)	31 (34.8%)	
Adequate physical activity				
No	343 (67.0%)	214 (62.4%)	129 (37.6%)	0.073
Yes	169 (33.0%)	119 (70.4%)	50 (29.6%)	
Career (year)				
	2.14 ± 1.39	2.19 ± 1.37	2.05 ± 1.44	0.272
Working hours (hours/week)				
≤ 40	73 (14.3%)	52 (71.2%)	21 (28.8%)	0.305
41~ 50	249 (48.6%)	166 (66.7%)	83 (33.3%)	
51~ 60	145 (28.3%)	86 (59.3%)	59 (40.7%)	
≥ 61	45 (8.8%)	29 (64.4%)	16 (35.6%)	
Job task				
Office	445 (86.9%)	284 (63.8%)	161 (36.2%)	0.136
Labor/Service	67 (13.1%)	49 (73.1%)	18 (26.9%)	
Education				
≤ College	417 (81.4%)	268 (64.3%)	149 (35.7%)	0.444
≥ Master degree	95 (18.6%)	65 (68.4%)	30 (31.6%)	
Marital status				
Unmarried/Divorced	384 (75.0%)	247 (64.3%)	137 (35.7%)	0.556
Married	128 (25.0%)	86 (67.2%)	42 (32.8%)	
Occupational Stress				
1st quartile	201 (39.3%)	150 (74.6%)	51 (25.4%)	< 0.001
2nd quartile	161 (31.4%)	104 (64.6%)	57 (35.4%)	
3rd quartile	97 (18.9%)	51 (52.6%)	46 (47.4%)	
4th quartile	53 (10.4%)	28 (52.8%)	25 (47.2%)	
Total score	36.68 ± 10.30	35.20 ± 10.56	39.45 ± 9.22	< 0.001
Occupational stress sub-domains score				
Job demand	46.50 ± 16.41	44.84 ± 16.49	49.58 ± 15.83	0.002
Insufficient job control	45.25 ± 14.11	44.02 ± 14.47	47.53 ± 13.15	0.007
Interpersonal conflict	29.56 ± 13.63	28.66 ± 14.57	31.22 ± 11.53	0.030
Job insecurity	30.14 ± 20.45	28.48 ± 20.43	33.24 ± 20.18	0.012
Organizational system	38.75 ± 14.74	37.29 ± 15.18	41.48 ± 13.51	0.002
Lack of reward	37.17 ± 13.73	35.20 ± 13.51	40.84 ± 13.41	< 0.001
Occupational climate	29.41 ± 15.96	27.88 ± 16.15	32.26 ± 15.22	0.003

Logistic regression analysis was used to calculate the OR of the risk for depression by occupational stress. Compared to subjects with the lowest 25% of occupational-stress scores, the OR for developing depression in the second, third, and fourth quartiles, respectively, was 1.612 (1.025–2.536), 2.653 (1.594–4.416) and 2.626 (1.404–4.911) in model I. After adjusting for general and work-related characteristics, the OR for deteriorating depression in third and fourth quartiles was 2.513 (1.459–4.331) and 2.730 (1.389–5.363) compared to first quartile.

When the OR of the risk for depression was calculated using changes in occupational stress as a continuous variable, the results showed that the OR for developing depression was 1.044 (1.024–1.064) whenever the total occupational stress score increased by 1 point over the year. After adjusting for the 2015 occupational stress score, the OR was 1.087 (1.058–1.117). In addition, increased occupational stress score of all 7 sub-domains significantly developed depression in model I. Job demand, insufficient job control, job insecurity, organizational system, and lack of reward were still positive influenced on developing depression in model 3 (Table 3).

**Table 3:** Adjusted odds ratios for depression deterioration by occupational stress level at baseline and occupational stress change among non-depressed group (n = 512)

	Model 1		Model 2		Model 3	
	OR	95% CI	OR	95% CI	OR	95% CI
Occupational stress level						
1st quartile	1.00	Reference	1.00	Reference		
2nd quartile	1.612*	1.025–2.536	1.583	0.977–2.564		
3rd quartile	2.653‡	1.594–4.416	2.513‡	1.459–4.331		
4th quartile	2.626†	1.404–4.911	2.730†	1.389–5.363		
Occupational stress change ^a^						
Total score	1.044‡	1.024–1.064	1.039‡	1.018–1.062	1.087‡	1.058–1.117
Job demand	1.018†	1.007–1.030	1.025‡	1.014–1.037	1.050‡	1.034–1.065
Insufficient job control	1.018†	1.005–1.031	1.016*	1.003–1.029	1.032‡	1.016–1.048
Interpersonal conflict	1.014*	1.000–1.028	0.999	0.987–1.011	1.008	0.993–1.023
Job insecurity	1.011*	1.002–1.021	1.006	0.998–1.015	1.015†	1.005–1.025
Organizational system	1.020†	1.007–1.033	1.018†	1.004–1.031	1.037‡	1.021–1.053
Lack of reward	1.032‡	1.017–1.047	1.007	0.995–1.019	1.028‡	1.013–1.043
Occupational climate	1.018†	1.003–1.030	1.016*	1.003–1.029	0.999	0.984–1.014

Model 1: Crude model

Model 2: Adjusted for sex, age, smoking history, heavy drinking, adequate physical activity, education, marital status, career, working hours, job task

Model 3: Model 2 + occupational stress score (total or sub-domain) at baseline

^a^Subtracted 2015 KOSS score from 2016 KOSS score

*p < 0.05, †*p* < 0.01, ‡*p* < 0.001

### Depressed group

In 2016, 394 (75.2%) of them remained in the depressed-maintained group, whereas 130 (24.8%) experienced an alleviation of depressive symptoms and moved to the depressed-alleviated group. Concerning the general characteristics, the subjects in this group tended to be males, non-smokers, not excessive drinkers, college graduates or lower, unmarried, and workers who did not engage in adequate physical activity. As for work-related characteristics, the subjects in this group tended to be white-collar workers and to work 51 to 60 h per week. The group’s mean age was 28.30 years and the mean length of service was 2.37 years. The proportion of subjects with occupational stress was highest in the fourth quartile, followed by the third, second, and first quartiles, unlike the non-depressed group, which had the highest proportion in the first quartile. The total mean score was 46.9 points in the depressed-maintained group and 45.9 points in the alleviated group, and no statistically significant differences were found between the depressed-maintained group and the depressed-alleviated group on any of the variables (Table 4).

**Table 4:** General characteristics of study subjects among depressed group at baseline

	Total Depressed group (*n* = 524, 100.0%)	Depressed-maintained group (*n* = 394, 75.2%)	Depressed-alleviated group (*n* = 130, 24.8%)	*p*-value
	N (%) or M ± SD	N (%) or M ± SD	N (%) or M ± SD	
Sex				
Male	415 (79.2%)	310 (74.7%)	105 (25.3%)	0.611
Female	109 (20.8%)	84 (77.1%)	25 (22.9%)	
Age (year)				
	28.30 ± 2.93	28.23 ± 2.96	28.51 ± 2.84	0.342
Smoking history				
Non smoker	292 (55.7%)	218 (74.7%)	74 (25.3%)	0.611
Past smoker	83 (15.8%)	60 (72.3%)	23 (27.7%)	
Current smoker	149 (28.4%)	116 (77.9%)	33 (22.1%)	
Heavy drinking				
No	424 (80.9%)	315 (74.3%)	109 (25.7%)	0.327
Yes	100 (19.1%)	79 (79.0%)	21 (21.0%)	
Adequate physical activity				
No	401 (76.5%)	302 (75.3%)	99 (24.7%)	0.908
Yes	123 (23.5%)	92 (74.8%)	31 (25.2%)	
Career (year)				
	2.37 ± 1.23	2.35 ± 1.25	2.45 ± 1.18	0.396
Working hours (hours/week)				
≤ 40	49 (9.4%)	37 (75.5%)	12 (24.5%)	0.525
41~ 50	183 (34.9%)	143 (78.1%)	40 (21.9%)	
51~ 60	206 (39.3%)	154 (74.8%)	52 (25.2%)	
≥ 61	86 (16.4%)	60 (69.8%)	26 (30.2%)	
Job task				
Office	441 (84.2%)	329 (74.6%)	112 (25.4%)	0.473
Labor/Service	83 (15.8%)	65 (78.3%)	18(21.7%)	
Education				
≤ College	423 (80.7%)	313 (74.0%)	110 (26.0%)	0.195
≥ Master degree	101 (19.3%)	81 (80.2%)	20 (19.8%)	
Marital status				
Unmarried/Divorced	409 (78.1%)	312 (76.3%)	97 (23.7%)	0.275
Married	115 (21.9%)	82 (71.3%)	33 (28.7%)	
Occupational Stress				
1st quartile	55 (10.5%)	42 (76.4%)	13 (23.6%)	0.834
2nd quartile	108 (20.6%)	78 (72.2%)	30 (27.8%)	
3rd quartile	154 (29.4%)	115 (74.7%)	39 (25.3%)	
4th quartile	207 (39.5%)	159 (76.8%)	48 (23.2%)	
Total score	46.63 ± 10.07	46.87 ± 10.39	45.92 ± 9.03	0.348
Occupational stress sub-domains score				
Job demand	57.67 ± 16.95	57.68 ± 16.92	57.63 ± 17.08	0.977
Insufficient job control	51.32 ± 15.13	51.65 ± 15.31	50.32 ± 14.58	0.386
Interpersonal conflict	37.70 ± 14.12	38.10 ± 14.38	36.50 ± 13.29	0.262
Job insecurity	40.94 ± 22.06	40.23 ± 22.93	43.08 ± 19.11	0.163
Organizational system	47.77 ± 17.29	48.20 ± 18.18	46.47 ± 14.26	0.324
Lack of reward	50.38 ± 16.01	51.38 ± 16.33	47.35 ± 14.68	0.013
Occupational climate	40.66 ± 16.79	40.86 ± 10.39	40.06 ± 15.84	0.639

Logistic regression analysis was used to calculate the OR for the alleviation of depression by occupational stress levels. When the second, third, and fourth quartiles were compared with the first quartile of occupational stress scores, no statistically significant difference was found for any of the comparisons.

However, when the OR for the alleviation of depression were estimated using changes in occupational stress as a continuous variable, the results showed that the OR for alleviating depression was 1.063 (1.038–1.090) when the occupational stress score decreased by 1 point over the year. After adjusting for the 2015 occupational stress, the OR was 1.095 (1.062–1.128). In addition, the ORs for alleviating depression were 1.033 (1.020–1.045), 1.030 (1.015–1.045), 1.025 (1.012–1.039) when job demand, organizational system, lack of reward scores decreased by 1 point respectively. Job demand, insufficient job control, interpersonal conflict, organizational system, and lack of reward were sub-domains that significantly influenced on alleviating depression in Model 3 (Table 5).

**Table 5:** Adjusted odds ratios for depression alleviation by occupational stress level at baseline and occupational stress change among depressed group (*n* = 524)

	Model 1		Model 2		Model 3	
	OR	95% CI	OR	95% CI	OR	95% CI
Occupational stress level						
1st quartile	1.025	0.509–2.066	1.190	0.576–2.459		
2nd quartile	1.274	0.749–2.166	1.413	0.811–2.461		
3rd quartile	1.123	0.691–1.826	1.216	0.739–1.999		
4th quartile	1.000	Reference	1.000	Reference		
Occupational stress change ^a^						
Total score	1.063‡	1.038–1.090	1.062‡	1.035–1.089	1.095‡	1.062–1.128
Job demand	1.033‡	1.020–1.045	1.033‡	1.020–1.046	1.053‡	1.036–1.071
Insufficient job control	1.013	0.999–1.028	1.013	0.998–1.028	1.023*	1.005–1.041
Interpersonal conflict	1.009	0.997–1.022	1.008	0.995–1.022	1.025†	1.007–1.042
Job insecurity	1.007	0.998–1.016	1.007	0.997–1.016	1.005	0.994–1.016
Organizational system	1.030‡	1.015–1.045	1.029‡	1.014–1.045	1.049‡	1.030–1.069
Lack of reward	1.025‡	1.012–1.039	1.025‡	1.011–1.039	1.057‡	1.037–1.077
Occupational climate	1.007	0.995–1.020	1.006	0.993–1.020	1.011	0.996–1.026

Model 1: Crude model

Model 2: Adjusted for sex, age, smoking history, heavy drinking, adequate physical activity, education, marital status, career, working hours, job task

Model 3: Model 2 + occupational stress score (total or sub-domain) at baseline

^a^Subtracted 2016 KOSS score from 2015 KOSS score

*p < 0.05, †*p* < 0.01, ‡*p* < 0.001

## Discussion

This prospective study is the first in South Korea to investigate depression risk by level of occupational stress using the Korean Occupational Stress Scale. It also examined changes in occupational stress and the relationship between occupational stress and depression among workers in an electronic appliance manufacturing company. The occupational stress was prospectively associated with the development of depressive symptoms. Increased occupational stress developed depressive symptoms, and reduced stress regulated depressive symptoms.

The results of this study showed that 524 of 1036 subjects were in the depressed group, accounting for 50.6% of all subjects. This figure was somewhat higher compared to a study of nurses using the WHO-5 Index, in which the proportion of those at risk for depression was 35.4% and a large-scale study of 50,032 subjects using data from the 3rd Korean Working Conditions Survey, in which 39.0% were at risk [18, 19]. However, a study of male white-collar workers in an automobile company found that 57.7% had depressive symptoms [10], which might be related to differences in type of occupation or the workplace atmosphere. All subjects in this study were young adults with less than 5 years of service. The results of this study are consistent with the tendency for people with shorter employment periods to have higher stress levels, as they adapt to environmental changes or the organizational culture [20, 21]. Meanwhile, inadequate physical activity and long working hours tended to associate with the depressed group. The non-depressed group had the first quartile occupational stress level most, followed by the second, third, and fourth quartiles, whereas the depressed group showed the opposite results. Likewise, stress measured by continuous variable was higher in the depressed group than non-depressed group in every domains. The results of this study showed a positive correlation between stress and depression, which is consistent with the results of previous cross-sectional studies [22].

A comparison statistics was performed after the non-depressed group was divided into the non-depressed-maintained group and the non-depressed-deteriorated group, while the depressed group was divided into the depressed-maintained group and the depressed-alleviated group. The results showed there were no statistically significant differences between the groups on the study’s measures, such as personal and work-related characteristics. This is because the study subjects consisted only of workers with similar backgrounds. However, there was a difference in occupational stress variables between the non-depressed-maintained group and the deteriorated group. As a result, the effect of occupational stress as the sole independent variable affecting depression was supported. In addition, sex, socioeconomic status, drinking, and physical activity, which were important confounding factors for stress and depression, were investigated [23–26], but no effects were found in this analysis.

The first quartile of occupational stress in the non-depressed group showed no significant difference from the second quartile after adjustment, but the incidence of depression reported high at the third and fourth quartile groups (2.51, 2.73, respectively). The quartile was used for assessing and managing occupational stress, and clinically, it is worthwhile to discuss the use of the median as a cut-off point for the identification of at-risk groups. Interestingly, a one-point increase in the occupational stress score over 1 year was found to increase the presence of depression by 8.7%, regardless of the baseline occupational stress score. Special attention should be paid to workers with steep increases in occupational stress scores in repeated stress tests, even if their current occupational stress score is low. Furthermore, we found that depressive symptom could be developed by reason of not only change of the total score of occupational stress but increased scores of any of the sub-domains. A cohort study conducted in Denmark also found that job insecurity in male workers increased the relative risk of depressive symptoms by 2.04 times [23], and a meta-analysis found that occupational stress increased the risk for depression by 1.77 times [27]. The finding of this study showing that increased stress caused depression and the deterioration of mental health is consistent with the results of prospective studies conducted in other counties using other stress scales [28–30].

On the other hand, no significant differences were found in occupational stress level and score as well as general and work-related characteristics between the depressed-maintained group and the depressed-alleviated group. Therefore, unlike the deteriorated group, the occupational stress level at baseline had no significant effect on the reduction of depression. However, when the occupational stress score decreased by 1 point over the year, the probability of moving to the non-depressed group increased by 6.3, 6.2% in Model 1, 2. After adjusting for the 2015 score, the probability increased further by 9.5%, indicating that the changes in stress had more pronounced effects on the recovery from depression than the current stress level. In terms of sub-items, a one-point decrease in job demand, organizational system, and lack of reward scores over 1 year reduced the presence of depression in Model 1, 2, respectively. In Model 3, the influence of these sub-domains to alleviate depression was stronger and decrease of insufficient job control and interpersonal conflict scores also reduced depression. The ORs for the alleviation of depression by the rest changes of job insecurity, occupational climate were greater than 1, although these were not significant. Eventually, decrease of any of sub-domains score could influence on depression positively in common with depression deterioration. The results of a study using an effort-reward imbalance model found that the improved E-R ratio score over 2 years was significantly associated with improvements in anxiety and depression, and the degree of change in score rather than the absolute score was more strongly associated with improvements in anxiety and depression, which is consistent with the results of this study [15].

Depression is closely related to the serotonin system and the cytokine-immune system. Psychological stress induces a deficiency of tryptophan, a precursor of serotonin, causing mood swings. At the same time, the body attempts to maintain balance by altering serotonin synthesis and the expression of various serotonin receptors. However, if chronic psychological stress persists, pro-inflammatory cytokines increase and the balance of anti-inflammatory cytokines is disturbed due to the compensatory mechanism, thereby resulting in depressive symptoms [31]. This is thought to be similar to the effects of occupational stress on depression.

The strengths of this study are as follows. First, occupational stress level and changes in occupational stress over 1 year were quantified through a prospective study design, and their associations with the risk for depression were analyzed. Most of the related studies conducted in South Korea have used cross-sectional designs. However, this study is noticeable because it was the first study to investigate the causal relationship between the risk for depression and stress. Second, the reliability of the survey instruments used in this study is another strength. The objectivity and validity of the Korean Occupational Stress Scale was confirmed in the course of its development and the WHO-5 Well-being Index has been validated as an effective screening tool for depression, which increases the reliability of the present study’s results [16, 32].

This study has limitations. First, the subjects consisted of workers with the same type of occupation who worked in the same workplace setting. Given that the subjects’ mean age was 28 years, they had less than 5 years of service, were mostly white-collar workers, and non-shift workers, it is difficult to generalize the results of this study to all workers. Second, length of follow-up time was somewhat short (1 year) as resulting improvements in health may take longer to develop [33]. Therefore, further studies are needed in the future.

## Conclusions

In conclusion, this study confirmed association between occupational stress and depressive symptom. In addition, we found that increased occupational stress predicted the presence of depressive symptoms, and that depressive symptoms could be controlled through stress reduction. Further studies are needed to expand the types of subjects to a wider variety of workers. The conduct and effects of stress intervention programs also require investigation.

## Abbreviations

CI: Confidence interval
OR: Odds ratio
SD: Standard deviation
